# Supplementary material for: Breaking down linguistic barriers: The radical impact of translanguaging on pre-service EFL teachers’ perspectives in Turkey
Source: PLoS One. 2024 Dec 23;19(12):e0315982. doi: 10.1371/journal.pone.0315982 (PMC11666009; doi:10.1371/journal.pone.0315982)
Supplement: Attachment — Submitted filename: Translanguaging.pdf [file pone.0315982.s003.pdf]

PLOS ONE

Breaking Down Linguistic Barriers: The Radical Impact of Translanguaging on Pre-Service EFL Teachers' Perspectives in Turkey

--Manuscript Draft--

**Manuscript Number:**	PONE-D-24-31275
**Article Type:**	Research Article
**Full Title:**	Breaking Down Linguistic Barriers: The Radical Impact of Translanguaging on Pre-Service EFL Teachers' Perspectives in Turkey
**Short Title:**	The Radical Impact of Translanguaging on Pre-Service EFL Teachers
**Corresponding Author:**	Ömer Gökhan Ulum<br>Mersin University: Mersin Universitesi<br>Yenişehir, Mersin TÜRKIYE
**Keywords:**	translanguaging; EFL; pre-service teachers; language education
**Abstract:**	This study examines the perceptions and practices of translanguaging among pre-service English as a Foreign Language (EFL) teachers in Turkey, employing a mixed-method research design with a phenomenological framework. The sample consists of 401 pre-service EFL teachers (243 female, 158 male) from the Education Faculty's English Language Teaching (ELT) Department at a state university during the 2024-2025 academic year. The study involved 45 preparatory class students, 49 first-year students, 129 second-year students, 100 third-year students, and 78 fourth-year students. Data collection included a rigorously developed 28-item Likert-scale questionnaire and semi-structured interviews with 120 participants. The questionnaire, refined through factor analysis, explored various dimensions of translanguaging, including its perceived benefits, challenges, and usage contexts. Quantitative data were analyzed using descriptive and inferential statistics, while qualitative data were examined through thematic analysis. The results indicate that translanguaging is largely perceived as a valuable and natural pedagogical strategy that alleviates anxiety, enhances confidence, and promotes active classroom participation. This research underscores the significance of integrating translanguaging in teacher education programs and highlights the need for further investigation into its diverse effects on language learning and teaching practices.
**Order of Authors:**	Ömer Gökhan Ulum
**Opposed Reviewers:**	
**Additional Information:**	
**Question**	**Response**
**Financial Disclosure**<br><br>Enter a financial disclosure statement that describes the sources of funding for the work included in this submission. Review the submission guidelines for detailed requirements. View published research articles from *PLOS ONE* for specific examples.<br><br>This statement is required for submission and **will appear in the published article** if the submission is accepted. Please make sure it is accurate.	The author(s) received no specific funding for this work.

Powered by Editorial Manager® and ProduXion Manager® from Aries Systems Corporation

**Funded studies**<br>Enter a statement with the following details:<br>• Initials of the authors who received each award<br>• Grant numbers awarded to each author<br>• The full name of each funder<br>• URL of each funder website<br>• Did the sponsors or funders play any role in the study design, data collection and analysis, decision to publish, or preparation of the manuscript?<br><br>Did you receive funding for this work?	
**Competing Interests**<br><br>Use the instructions below to enter a competing interest statement for this submission. On behalf of all authors, disclose any competing interests that could be perceived to bias this work—acknowledging all financial support and any other relevant financial or non-financial competing interests.<br><br>This statement is required for submission and **will appear in the published article** if the submission is accepted. Please make sure it is accurate and that any funding sources listed in your Funding Information later in the submission form are also declared in your Financial Disclosure statement.<br><br>View published research articles from *PLOS ONE* for specific examples.	The authors have declared that no competing interests exist.

Powered by Editorial Manager® and ProduXion Manager® from Aries Systems Corporation

**NO authors have competing interests**<br><br>Enter: *The authors have declared that no competing interests exist.*<br><br>**Authors with competing interests**<br><br>Enter competing interest details beginning with this statement:<br><br>*I have read the journal's policy and the authors of this manuscript have the following competing interests: [insert competing interests here]*<br><br>* typeset	
**Ethics Statement**<br><br>Enter an ethics statement for this submission. This statement is required if the study involved:<br><br>• Human participants<br>• Human specimens or tissue<br>• Vertebrate animals or cephalopods<br>• Vertebrate embryos or tissues<br>• Field research<br><br>Write "N/A" if the submission does not require an ethics statement.<br><br>General guidance is provided below. Consult the submission guidelines for detailed instructions. **Make sure that all information entered here is included in the Methods section of the manuscript.**	The study titled "Breaking Down Linguistic Barriers: The Radical Impact of Translanguaging on Pre-Service EFL Teachers' Perspectives in Turkey" by Assoc. Prof. Dr. Ömer Gökhan Ulum has been reviewed by the Mersin University Social and Human Sciences Ethics Committee. The study was found to be ethically appropriate in the decision dated July 11, 2024, with Decision Number 249. Additionally, written informed consent was provided by all participants.

Powered by Editorial Manager® and ProduXion Manager® from Aries Systems Corporation

**Format for specific study types**<br><br>**Human Subject Research (involving human participants and/or tissue)**<br>• Give the name of the institutional review board or ethics committee that approved the study<br>• Include the approval number and/or a statement indicating approval of this research<br>• Indicate the form of consent obtained (written/oral) or the reason that consent was not obtained (e.g. the data were analyzed anonymously)<br><br>**Animal Research (involving vertebrate animals, embryos or tissues)**<br>• Provide the name of the Institutional Animal Care and Use Committee (IACUC) or other relevant ethics board that reviewed the study protocol, and indicate whether they approved this research or granted a formal waiver of ethical approval<br>• Include an approval number if one was obtained<br>• If the study involved *non-human primates*, add *additional details* about animal welfare and steps taken to ameliorate suffering<br>• If anesthesia, euthanasia, or any kind of animal sacrifice is part of the study, include briefly which substances and/or methods were applied<br><br>**Field Research**<br><br>Include the following details if this study involves the collection of plant, animal, or other materials from a natural setting:<br>• Field permit number<br>• Name of the institution or relevant body that granted permission	
**Data Availability**<br><br>Authors are required to make all data underlying the findings described fully available, without restriction, and from the time of publication. PLOS allows rare exceptions to address legal and ethical concerns. See the PLOS Data Policy and FAQ for detailed information.	Yes - all data are fully available without restriction

A Data Availability Statement describing where the data can be found is required at submission. Your answers to this question constitute the Data Availability Statement and **will be published in the article**, if accepted.<br><br>**Important:** Stating 'data available on request from the author' is not sufficient. If your data are only available upon request, select 'No' for the first question and explain your exceptional situation in the text box.<br><br>Do the authors confirm that all data underlying the findings described in their manuscript are fully available without restriction?	
**Describe where the data may be found in full sentences. If you are copying our sample text, replace any instances of XXX with the appropriate details.**<br><br>• If the data are **held or will be held in a public repository**, include URLs, accession numbers or DOIs. If this information will only be available after acceptance, indicate this by ticking the box below. For example: *All XXX files are available from the XXX database (accession number(s) XXX, XXX.*).<br>• If the data are all contained **within the manuscript and/or Supporting Information files**, enter the following: *All relevant data are within the manuscript and its Supporting Information files.*<br>• If neither of these applies but you are able to provide **details of access elsewhere**, with or without limitations, please do so. For example:<br><br>*Data cannot be shared publicly because of [XXX]. Data are available from the XXX Institutional Data Access / Ethics Committee (contact via XXX) for researchers who meet the criteria for access to confidential data.*<br><br>*The data underlying the results presented in the study are available from (include the name of the third party*	All relevant data are within the manuscript and its Supporting Information files.

and contact information or URL).<br>• This text is appropriate if the data are owned by a third party and authors do not have permission to share the data.<br><br>* typeset	
Additional data availability information:	Tick here if the URLs/accession numbers/DOIs will be available only after acceptance of the manuscript for publication so that we can ensure their inclusion before publication.

Powered by Editorial Manager® and ProduXion Manager® from Aries Systems Corporation

Dear Editor,

This is a manuscript titled "Breaking Down Linguistic Barriers: The Radical Impact of Translanguaging on Pre-Service EFL Teachers' Perspectives in Turkey." This paper is new. The entire paper or any part of it has not been published or accepted elsewhere. It has not been submitted to any other journals.

This paper is of great significance to the learning and teaching of EFL. First, it examines the perceptions and practices of translanguaging among pre-service EFL teachers, which can deepen the understanding of translanguaging as a pedagogical strategy. This discovery provides empirical evidence for its benefits, such as reducing anxiety, boosting confidence, and promoting active classroom participation. It also enriches the theory of language teaching and learning by demonstrating how translanguaging can be effectively integrated into teacher education programs.

Second, the findings reveal the positive impact of translanguaging on language learning outcomes, which provides new teaching tools and strategies for EFL educators. This helps teachers better utilize translanguaging to assist in English language instruction and improve students' learning experiences.

Third, this study offers a new perspective and tool for EFL teaching, contributing to innovation and development in language education. It underscores the importance of translanguaging in fostering a more inclusive and effective learning environment.

Last, the research promotes the integration and innovation of educational practices, advancing the modernization and development of language teaching methodologies.

This paper is also one of the multiple articles related to EFL learning and teaching during the author’s years of teaching English. We believe that readers of your journal may be interested in this paper because it describes related studies of translanguaging in higher education.

I have reviewed the manuscript and approved the submission to your journal, and I will not submit it to other journals until the editors and experts make a final decision. Thank you very much for your attention and consideration.

Yours sincerely,

Associate Professor Dr. Ömer Gökhan Ulum

Manuscript
Click here to access/download;Manuscript;Breaking Down Linguistic Barriers.edited (1).docx

Title Page

Breaking Down Linguistic Barriers: The Radical Impact of Translanguaging on Pre-Service EFL Teachers' Perspectives in Turkey

Assoc. Prof. Dr. Ömer Gökhan Ulum
Mersin University, Education Faculty, Department of English Language Education, Turkey

Current Address: Mersin University, Education Faculty, Department of English Language Education, Turkey

Corresponding Author: Assoc. Prof. Dr. Ömer Gökhan Ulum
e-mail: omergokhanulum@gmail.com

Conflict of Interest Statement
I, as the researcher, declare that no conflict of interests exists.

Funding Statement
I, as the researcher, received no funding for this study.

Ethical Statement
The informants who agreed to participate in the study completed a written informed consent form, ensuring their voluntary and informed participation.

Author contributions

Assoc. Prof. Dr. Ömer Gökhan Ulum was responsible for conducting every phase of the research.

Abstract

This study examines the perceptions and practices of translanguaging among pre-service English as a Foreign Language (EFL) teachers in Turkey, employing a mixed-method research design with a phenomenological framework. The sample consists of 401 pre-service EFL teachers ~~(243 female, 158 male)~~ from the Education Faculty's English Language Teaching (ELT) Department at a state university during the 2024-2025 academic year. ~~The study involved 45 preparatory class students, 49 first-year students, 129 second-year students, 100 third-year students, and 78 fourth-year students.~~ Data collection included a rigorously developed 28-item Likert-scale questionnaire and semi-structured interviews ~~with 120 participants~~. The questionnaire, refined through factor analysis, explored various dimensions of translanguaging, including its perceived benefits, challenges, and usage contexts. Quantitative data were analyzed using descriptive and inferential statistics, while qualitative data were examined through thematic analysis. The results indicate that translanguaging is largely perceived as a valuable and natural pedagogical strategy that alleviates anxiety, enhances confidence, and promotes active classroom participation. This research underscores the significance of integrating translanguaging in teacher education programs and highlights the need for further investigation into its diverse effects on language learning and teaching practices.

Keywords: translanguaging, EFL, pre-service teachers, language education

1. Introduction

In recent years, the concept of translanguaging has garnered significant attention in the field of language education (Bonacina-Pugh et al., 2021; Fang et al., 2022; Liu & Fang, 2022; Leung & Valdés, 2019; Makarova et al., 2023; Prilutskaya, 2021). Translanguaging, a strategy involving multiple languages within a classroom setting, allows students and teachers to draw on their full linguistic repertoire during lessons (Bonacina-Pugh et al., 2021; García & Kleyn,

2016; Nagy, 2018). This approach recognizes and utilizes the languages students are already familiar with (García & Seltzer, 2016), such as Turkish, Azerbaijani, Kurdish, Arabic, and English, throughout various stages of instruction. The strategy of translanguaging in English lessons can be seen from multiple perspectives (Ticheloven et al., 2021). Leung and Valdés (2019) argue that it is a natural and beneficial practice that supports language learning by reducing anxiety (Back et al., 2020), increasing confidence (Song et al., 2022), and facilitating comprehension (Makalela, 2015). On the other hand, translanguaging is also viewed as a consequence of inadequate English language proficiency, whether in terms of linguistic skills, speaking abilities, or confidence in using English exclusively (Jaspers, 2018). The primary aim of this research is to explore the perceptions of pre-service English teachers regarding the use of translanguaging in English lessons. Specifically, the study will investigate whether translanguaging is perceived as a useful pedagogical tool or a necessity born out of linguistic deficiencies. Furthermore, the research will examine the potential benefits of translanguaging for enhancing participation, motivation, effective communication, creative thinking, and critical reflection among students.

The following research questions guide this investigation:

Main Research Question

How do various factors influence translanguaging among pre-service English as a Foreign Language (EFL) teachers in state university settings?

Sub-Research Questions

1. To what extent do gender differences impact the utilization of translanguaging in EFL environments?
2. How does the application of translanguaging vary across different classes within EFL settings?

3. What are the general perceptions of pre-service EFL teachers at a state university regarding the translanguaging strategy in EFL contexts?
4. How do pre-service EFL teachers at a state university perceive the application of translanguaging within specific EFL contexts?
5. What are the perceived benefits of translanguaging according to pre-service EFL teachers at a state university?

2. Background of the Study

Translanguaging, a concept rooted in bilingual education (Bonacina-Pugh et al., 2021), has evolved as a critical pedagogical strategy in multilingual classrooms (Creese & Blackledge, 2010). Originating from the work of Welsh scholars in the 1980s, translanguaging involves the fluid and dynamic use of multiple languages to facilitate learning and communication (Jones et al., 2017; Lewis et al., 2012). It challenges the traditional view of language separation (Jonsson, 2017; Palmer, 2014) and promotes the integration of students' linguistic resources in a cohesive and meaningful manner (Cenoz & Gorter, 2020; Sobkowiak, 2022). In the context of English language teaching, translanguaging has gained prominence as educators recognize the diverse linguistic backgrounds of their students (Barahona, 2020; Ossa Parra & Proctor, 2021). Turkey, characterized by its rich linguistic diversity, presents a unique landscape for exploring the implications of translanguaging in English language classrooms (Ataş, 2023; Yilmaz, 2021). For students who speak Turkish, Azerbaijani, Kurdish, and Arabic, English language instruction in Turkey provides a fertile ground for implementing and studying translanguaging practices (Koçer, 2023). The theoretical foundations of translanguaging are supported by several educational theories, including Vygotsky's sociocultural theory, which emphasizes the role of social interaction and cultural tools in learning (Duarte, 2019; Smith & Robertson, 2020), and Cummins' threshold hypothesis, which suggests that a certain level of proficiency in both languages is necessary for cognitive benefits to manifest (Villacañas de Castro, 2016;

Cummins, 2021). Translanguaging aligns with these theories by leveraging students' existing linguistic competencies to enhance their learning experiences. Empirical studies on translanguaging have demonstrated its potential benefits in various educational settings (Bonacina-Pugh et al., 2021; Carstens, 2016; Prilutskaya, 2021). For instance, research has shown that translanguaging can reduce language anxiety (Nursanti, 2021), increase student participation (Shawaqfeh et al., 2024), and foster a deeper understanding of the subject matter (Robillos, 2023). It also allows students to express complex ideas and engage in higher-order thinking using their full linguistic repertoire (Khair et al., 2020). Additionally, translanguaging has been found to support the maintenance and development of students' home languages, contributing to their linguistic and cultural identity (Carstens, 2016; Cenoz & Gorter, 2020; Holdway & Hitchcock, 2018). Despite its advantages, translanguaging presents challenges and raises concerns (Galante, 2020; Vaish, 2019). Some educators and stakeholders worry that it might hinder the development of English proficiency, particularly if students rely too heavily on their first languages (Fang & Liu, 2020). There are also concerns about the feasibility of implementing translanguaging practices in classrooms with varying levels of language proficiency among students (Liu & Fang, 2022).

In Turkey, the adoption of translanguaging in English language classrooms is influenced by several factors (Karabulut & Dollar, 2022; Yilmaz, 2021; Yuvayapan, 2019), including teachers' beliefs (Öztürk et al., 2023; Yuvayapan, 2019), instructional practices (Genc et al., 2023), and institutional policies (Genc et al., 2023; Yuvayapan, 2019). Teachers' perceptions of translanguaging play a crucial role in its implementation (Yasar et al., 2022; Yuvayapan, 2019). While some teachers view it as a valuable strategy to support learning (Ticheloven et al., 2021; Yuvayapan, 2019), others perceive it as a sign of linguistic deficiency or lack of confidence in using English (Fang & Liu, 2020; Mwinda & Van der Walt, 2015).

This study seeks to investigate these diverse perspectives and contribute to the ongoing discourse on translanguaging in English language education. By examining the perceptions of both teachers and students, the research aims to provide a comprehensive understanding of the role of translanguaging in English language classrooms in Turkey. The findings will offer insights into how translanguaging can be effectively integrated into language teaching practices, addressing its potential benefits and challenges. Ultimately, this study aims to inform educators, policymakers, and researchers about the practical implications of translanguaging, promoting its thoughtful and strategic use to enhance language learning outcomes and support the linguistic and cultural diversity of students in Turkey.

3. Significance of the Study

The significance of this study on translanguaging in English language classrooms in Turkey is multifaceted, addressing several critical areas in language education and broader educational practices. First and foremost, this study contributes to understanding how translanguaging can enhance language learning. By leveraging students' full linguistic repertoire, translanguaging can deepen comprehension, facilitate learning complex concepts, and promote critical thinking. This research will provide empirical evidence on the effectiveness of translanguaging in improving English proficiency, thus offering valuable insights for language educators seeking to adopt more inclusive and effective teaching strategies. In a country as linguistically diverse as Turkey, promoting multilingualism is essential. This study highlights the importance of maintaining and valuing students' home languages while learning English. The findings will underscore the role of translanguaging in supporting linguistic diversity, helping educators foster an environment where multiple languages are seen as assets rather than barriers. This approach aligns with global educational trends that emphasize the value of multilingualism in fostering cognitive, social, and cultural development. Teacher perceptions and practices play a crucial role in successfully implementing translanguaging. By exploring the attitudes and

beliefs of English teachers towards translanguaging, this study will identify the factors that influence their willingness and ability to integrate this strategy into their teaching. Understanding these factors will enable the development of targeted professional development programs and resources that support teachers in effectively utilizing translanguaging to enhance their instructional practices. Language anxiety is a significant barrier to language acquisition. This study will examine how translanguaging can reduce anxiety and increase students' confidence in using English. By providing a supportive environment where students can draw on their linguistic strengths, translanguaging can help mitigate the stress associated with learning a new language. The insights from this research will be valuable for educators and policymakers aiming to create more inclusive and supportive language learning environments. The findings of this study have important implications for educational policy. Policymakers will better understand the benefits and challenges of translanguaging, informing decisions related to language education curricula and instructional strategies. By highlighting the practical benefits of translanguaging, this study can advocate for policies that support multilingual education and recognize the linguistic rights of students. Finally, this study significantly contributes to the academic literature on translanguaging and multilingual education. It comprehensively analyses the perceptions and practices surrounding translanguaging in a specific context, enriching the global discourse on language education. The research findings will serve as a valuable resource for scholars, educators, and students interested in the intersections of language, culture, and education. In summary, this study is significant because it explores the potential of translanguaging to enhance language learning, support multilingualism, inform teacher practices, reduce language anxiety, and shape educational policies. By addressing these areas, the research aims to promote a more inclusive and effective approach to language education that values and leverages the linguistic diversity of students in Turkey.

4. Methodology

4.1. Research Design

This study employs a mixed-method research design, integrating both quantitative and qualitative approaches to comprehensively understand the perceptions and practices surrounding translanguaging in English language classrooms. Specifically, the research follows a phenomenological approach to explore the lived experiences and perspectives of pre-service English as a Foreign Language (EFL) teachers at a state university in Turkey.

4.2. Participants

This study's participants are 401 pre-service EFL teachers enrolled in the Education Faculty, English Language Teaching (ELT) Department at a state university in Turkey for the 2024-2025 academic year. The participants include 45 preparatory class students, 49 first-year students, 129 second-year students, 100 third-year students, and 78 fourth-year students. The sample comprises 243 female and 158 male participants, representing the student body differently.

4.3. Data Collection Instruments

4.3.1. Questionnaire

A questionnaire was developed to collect quantitative data on the perceptions of pre-service EFL teachers regarding translanguaging. The development of the questionnaire involved a rigorous process, including a factor analysis to ensure the reliability and validity of the instrument. The questionnaire addressed various aspects of translanguaging, such as its perceived benefits, challenges, and the contexts in which it is most frequently used. The factor analysis helped to identify key dimensions of translanguaging perceptions, which were then used to structure the questionnaire. The final version of the questionnaire consisted of 34 items,

rated on a Likert scale from 1 (strongly disagree) to 5 (strongly agree), covering themes such as naturalness, usefulness, anxiety reduction, confidence building, and perceived necessity of translanguaging.

The Kaiser-Mayer-Olkin value was computed to be 0.865 ($X^2 = 7394.731$, df = 561, $p < .001$), indicating that the sample size was adequate for a factor analysis. Therefore, multiple-factor analyses were run on the data until no item was loaded into multiple factors, as indicated by a loading difference smaller than 0.10. As a result, items B27, B26, A17, A11, B21 and A12 were removed, finalizing the scale. As a result, items B27, B26, A17, A11, B21, and A12 were removed, finalizing the scale. The final group of items explained 71.052% of the variance in the data with 28 items and eight factors. The factors and their respective items are as follows: Factor 1 comprises items A7, A8, A2, A10, A9, and A1, with item loadings of 0.829, 0.761, 0.631, 0.597, 0.537, and 0.486, respectively. Factor 2 includes items C33, C34, C32, and C31, with loadings of 0.912, 0.839, 0.818, and 0.751. Factor 3 consists of items B24, B22, B23, and B25, with loadings of 0.947, 0.708, 0.675, and 0.606. Factor 4 contains items A14, A15, and A16, with loadings of 0.913, 0.892, and 0.696. Factor 5 comprises items B28, C30, B20, and C29, with loadings of 0.77, 0.744, 0.644, and 0.621. Factor 6 comprises items A4 and A3, with loadings of 0.986 and 0.727. Factor 7 includes items A6 and A5, with loadings of 0.859 and 0.792. Lastly, Factor 8 consists of items B18 and B19, with loadings of 0.992 and 0.714. These factors collectively explain the majority of the variance in the data, contributing to the robustness and reliability of the final scale. Factor loadings are presented below in Table 1.

Table 1. Factor Loadings

Item	1	2	3	4	5	6	7	8
A7	0.829	0.431	0.431	0.308	0.293	-0.082	-0.072	0.478
A8	0.761	0.327	0.311	0.25	0.352	-0.112	0.046	0.363

A2	0.631	0.343	0.351	0.327	0.317	-0.028	-0.094	0.224
A10	0.597	0.353	0.272	0.266	0.249	-0.056	0.138	0.355
A9	0.537	0.275	0.196	0.32	0.331	-0.017	0.227	0.315
A1	0.486	0.243	0.18	0.211	0.304	0.013	0.063	0.221
C33	0.426	0.912	0.572	0.458	0.364	-0.094	-0.09	0.445
C34	0.402	0.839	0.511	0.443	0.356	-0.062	-0.065	0.379
C32	0.445	0.818	0.517	0.397	0.586	-0.106	-0.16	0.358
C31	0.393	0.751	0.543	0.407	0.523	-0.058	-0.085	0.373
B24	0.356	0.533	0.947	0.394	0.259	0.033	-0.071	0.304
B22	0.522	0.607	0.708	0.506	0.313	-0.063	-0.059	0.478
B23	0.314	0.434	0.675	0.322	0.277	-0.057	-0.004	0.317
B25	0.358	0.497	0.606	0.295	0.251	-0.031	-0.021	0.405
A14	0.428	0.485	0.43	0.913	0.321	-0.009	-0.063	0.27
A15	0.331	0.434	0.441	0.892	0.39	0.012	-0.089	0.234
A16	0.304	0.422	0.313	0.696	0.364	-0.005	-0.202	0.152
B28	0.43	0.378	0.262	0.314	0.77	-0.111	0.045	0.267
C30	0.385	0.589	0.379	0.378	0.744	-0.197	-0.029	0.284
B20	0.468	0.287	0.224	0.21	0.644	-0.036	0.088	0.282
C29	0.2	0.327	0.213	0.309	0.621	-0.089	0.036	0.204
A4	-0.016	-0.066	-0.054	0.008	-0.064	0.986	0.573	-0.055
A3	-0.05	-0.122	-0.027	-0.062	-0.183	0.727	0.458	-0.023
A13	-0.071	0.02	0.076	0.15	-0.054	0.292	0.071	0.043
A6	0.098	-0.05	-0.027	-0.094	0.06	0.457	0.859	0.109
A5	-0.008	-0.12	-0.057	-0.114	0.039	0.518	0.792	-0.012
B18	0.447	0.438	0.413	0.261	0.324	-0.04	0.08	0.992

B19	0.516	0.436	0.348	0.206	0.254	-0.074	0.068	0.714

The reliability coefficient for the scale was found to be .888. The coefficients were .747 for A, .816 for B and .864 for C. The data was also seen to have been normally distributed in the scale and all its subscales. The skewness and kurtosis values were between -.709 and .399, indicating normal distributions. These values are presented in Table 2.

Table 2. Skewness and Kurtosis Values

Scale	Skewness	SE	Kurtosis	SE
scale_mean	-.589	.122	.234	.243
A_mean	-.068	.122	.399	.243
B_Mean	-.323	.122	-.491	.243
C_Mean	-.709	.122	-.171	.243

Based on the normal distributions, gender-based means were compared using an independent samples t-test. For class comparisons, one-way ANOVAs were used with Tukey post hoc analysis. However, LSD posthoc analysis was used for the A subscale since the ANOVA was significant, but Tukey posthoc tests did not produce any significant difference across groups.

4.3.2. Semi-Structured Interviews

To complement the quantitative data, semi-structured interviews were conducted with a subset of 120 pre-service EFL teachers. The interviews were designed by the researcher and aimed to delve deeper into the phenomenological aspects of translanguaging. The semi-structured format allowed for flexibility in exploring participants' experiences, attitudes, and practices related to translanguaging in their teaching. The interview questions were developed based on the questionnaire's findings and the translanguaging literature. Key topics included the participants'

experiences with translanguaging, their perceived advantages and disadvantages of using multiple languages in the classroom, and specific instances where translanguaging was used effectively or ineffectively.

4.3.3. Data Collection Procedure

The data collection process was carried out in two phases:

4.3.3.1. Quantitative Phase: The questionnaire was distributed to 401 pre-service EFL teachers at the state university. Participants completed the questionnaire during scheduled class sessions to ensure a high response rate and the integrity of the data collection process.

4.3.3.2. Qualitative Phase: 120 participants were selected for in-depth interviews following the quantitative phase. These participants were chosen based on their responses to the questionnaire, ensuring a diverse representation of perspectives. The interviews were conducted in a quiet and comfortable setting, each lasting approximately 25-30 minutes. All interviews were audio-recorded with the participant's consent and subsequently transcribed for analysis.

5. Data Analysis

5.1. Quantitative Data

The quantitative data from the questionnaire were analyzed using descriptive and inferential statistics. Descriptive statistics provided an overview of the general trends and patterns in the participants' perceptions of translanguaging. Factor analysis was used to confirm the underlying dimensions of the questionnaire items. Inferential statistics, such as t-tests and ANOVA, were employed to examine differences in perceptions based on demographic variables such as academic year, gender, and prior experience with multilingual education.

5.2. Qualitative Data

The qualitative data from the semi-structured interviews were analyzed using thematic analysis. The transcribed interviews were coded and categorized into themes that emerged from the data. This process involved multiple rounds of coding to ensure the accuracy and depth of the analysis. The themes were then compared and contrasted with the quantitative findings to understand the participants' perceptions and experiences with translanguaging comprehensively.

5.3. Ethical Considerations

The present study has been reviewed by the Mersin University Social and Human Sciences Ethics Committee. The study was found to be ethically appropriate in the decision dated July 11, 2024, with Decision Number 249. The recruitment period for the data collection were between July 11-20, 2024. Additionally, written informed consent was provided by all participants. Participants were assured of the confidentiality and anonymity of their responses, and they were informed of their right to withdraw from the study at any time without any consequences.

6. Findings and Results

6.1. Questionnaire Results

Gender comparisons are presented in Table 3.

Table 3. Gender Comparisons

Scale	Gender	N	M	SD	t	df	p
scale_mean	female	243	3.588	0.561	2.791	399	.006
	male	158	3.425	0.584			
A_mean	female	243	3.464	0.530	2.017	399	.044
	male	158	3.351	0.574			

B_Mean	female	243	3.603	0.790	2.690	399	.007
	male	158	3.388	0.776			
C_Mean	female	243	3.855	0.826	2.348	399	.019
	male	158	3.648	0.922			

All comparisons were significant according to the t-test results ($p < .05$). The results showed that the mean values for the male participants were significantly lower than those for the female participants in the scale and all its subscales. The descriptive results divided by classes and their ANOVA comparisons are given in Tables 4 and 5.

Table 4. Descriptive Results for Class Groups

Scale	Group	N	M	SD
scale_mean	prep	45	3.507	0.773
	1	49	3.526	0.737
	2	129	3.470	0.532
	3	100	3.431	0.506
	4	78	3.739	0.418
	Total	401	3.524	0.575
A_mean	prep	45	3.530	0.792
	1	49	3.520	0.749
	2	129	3.341	0.441
	3	100	3.331	0.470
	4	78	3.538	0.454
	Total	401	3.420	0.550
B_Mean	prep	45	3.478	1.009

	1	49	3.497	0.969
	2	129	3.517	0.822
	3	100	3.349	0.665
	4	78	3.776	0.531
	Total	401	3.518	0.791
C_Mean	prep	45	3.493	1.038
	1	49	3.575	0.998
	2	129	3.711	0.854
	3	100	3.777	0.746
	4	78	4.160	0.732
	Total	401	3.774	0.870

Table 5. ANOVA Results

Scale	Comparison	SS	df	MS	F	p	Differences
scale_mean	Between Groups	4.851	4	1.213	3.772	.005	4 > 3, 2 (p < .01)
	Within Groups	127.312	396	0.321			
	Total	132.163	400				
A_mean	Between Groups	3.730	4	0.932	3.152	.014	4 > 3, 2 (p < .01)
							prep > 2, 3 (p < .05)
	Within Groups	117.157	396	0.296			
	Total	120.887	400				1 > 2, 3 (p < .05)

B_Mean	Between Groups	8.136	4	2.034	3.329	.011	4 > 3 (p < .01)
	Within Groups	241.963	396	0.611			
	Total	250.099	400				
C_Mean	Between Groups	17.664	4	4.416	6.134	< .001	4 > 3 (p < .05)
	Within Groups	285.067	396	0.720			4 > 2, 1 (p < .01)
	Total	302.730	400				4 > prep (p < .001)

As seen in Table 4 and Table 5, there were significant differences in the scale and all its subscales ($p < .05$). Post hoc tests showed that 4th-year participants had higher means than 3rd-year and 2nd-year participants in scale total and subscale A, 3rd-year participants in subscale B and all other participants in subscale C ($p < .05$). In addition, prep and 1st-year participants were seen to have significantly higher means than 2nd and 3rd-year participants in subscale A ($p < .05$).

Table 6. Translanguaging Beliefs of Pre-service EFL teachers

Items	N	Minimum	Maximum	Mean	Std. Deviation
A1	401	1.00	5.00	3.9501	1.15651
A2	401	1.00	5.00	3.7681	.96363
A9	401	1.00	5.00	3.7307	1.01601
A13	401	1.00	5.00	3.5212	1.11138
A10	401	1.00	5.00	3.5137	1.16423
A14	401	1.00	5.00	3.4489	1.15022

A12	401	1.00	5.00	3.4464	1.15444
A8	401	1.00	5.00	3.3042	1.13455
A7	401	1.00	5.00	3.2868	1.15544
A6	401	1.00	5.00	3.2868	1.17900
A4	401	1.00	5.00	3.2818	1.16529
A3	401	1.00	5.00	3.1272	1.16029
A11	401	1.00	5.00	3.1247	1.19349
A5	401	1.00	5.00	3.0848	1.19908
Valid N (listwise)	401				

The descriptive statistics indicate that pre-service EFL teachers generally have a positive perception of translanguaging in English language classes. The highest agreement was observed for the view that translanguaging is a natural practice (mean = 3.9501, SD = 1.15651) and a beneficial strategy (mean = 3.7681, SD = 0.96363). Teachers also believe that translanguaging helps reduce anxiety (mean = 3.7307, SD = 1.01601) and boosts confidence (mean = 3.5137, SD = 1.16423) when using English. There is a positive attitude towards using different languages in the classroom (mean = 3.4464, SD = 1.15444), with support for preserving these languages (mean = 3.5212, SD = 1.11138) and viewing their use as a part of human rights (mean = 3.4489, SD = 1.15022). However, there is less agreement that translanguaging indicates deficiencies in English language skills, such as knowledge (mean = 3.1272, SD = 1.16029), speaking ability (mean = 3.2818, SD = 1.16529), confidence (mean = 3.0848, SD = 1.19908), and anxiety (mean = 3.2868, SD = 1.17900). Additionally, there is moderate agreement that translanguaging is necessary for learning English (mean = 3.2868, SD = 1.15544) and facilitates this process (mean = 3.3042, SD = 1.13455). The lowest agreement was for the perception that translanguaging disadvantages monolingual students (mean = 3.1247, SD = 1.19349).

Table 7. The Usage of Translanguaging Strategies by Pre-service EFL Teachers in Various Instructional Contexts

Items	N	Minimum	Maximum	Mean	Std. Deviation
B17	401	1.00	5.00	4.0748	.99468
B22	401	1.00	5.00	4.0599	.93883
B18	401	1.00	5.00	3.4264	1.28460
B21	401	1.00	5.00	3.3890	1.15900
B15	401	1.00	5.00	3.3666	1.39742
B19	401	1.00	5.00	3.3541	1.18080
B16	401	1.00	5.00	3.3142	1.22107
B20	401	1.00	5.00	3.1621	1.31763
Valid N (listwise)	401				

The descriptive statistics for English teachers using translanguaging strategies in different instructional contexts reveal interesting insights. The highest agreement among the pre-service EFL teachers was for using translanguaging to give instructions (mean = 4.0748, SD = 0.99468) and to explain concepts (mean = 4.0599, SD = 0.93883). There was moderate agreement for using translanguaging to help students with sufficient academic achievement (mean = 3.4264, SD = 1.28460), to develop foreign language skills (mean = 3.3890, SD = 1.15900), and to explain topics (mean = 3.3666, SD = 1.39742). Similarly, teachers moderately agreed on using translanguaging to develop high-achieving students further (mean = 3.3541, SD = 1.18080) and to conduct research related to the topic (mean = 3.3142, SD = 1.22107). The lowest agreement was for using translanguaging to support students when they face difficulties (mean = 3.1621, SD = 1.31763). These results suggest that while translanguaging is highly valued for

instructional clarity and student guidance, its use for supporting struggling students and enhancing academic performance is moderately beneficial.

Table 8. The Benefits of Translanguaging Strategies in English Learning Contexts

Items	N	Minimum	Maximum	Mean	Std. Deviation
C24	401	1.00	5.00	4.0150	1.01969
C23	401	1.00	5.00	4.0125	1.13681
C27	401	1.00	5.00	3.7382	1.11524
C26	401	1.00	5.00	3.7207	1.13216
C28	401	1.00	5.00	3.6833	1.14103
C25	401	1.00	5.00	3.4713	1.21441
Valid N (listwise)	401				

The descriptive statistics for the benefits of translanguaging strategies English learners use in various contexts indicate strong agreement on several aspects. The highest mean scores were observed for increasing participation (mean = 4.0150, SD = 1.01969) and motivation (mean = 4.0125, SD = 1.13681). Moderate agreement was found for using translanguaging in debates (mean = 3.7382, SD = 1.11524), ensuring effective communication (mean = 3.7207, SD = 1.13216), and fostering creative thinking (mean = 3.6833, SD = 1.14103). The lowest agreement was for using translanguaging to enhance critical and reflective thinking (mean = 3.4713, SD = 1.21441). These results suggest that translanguaging is particularly valued for boosting student participation and motivation. At the same time, its perceived effectiveness in promoting higher-order thinking skills is comparatively lower.

6.2. Interview Results

The following table represents the various aspects of translanguaging perceived by pre-service English as a Foreign Language (EFL) teachers, including their general attitudes towards its practice, personal usage patterns, intentionality, and its impact on classroom environments and language acquisition. The data highlights translanguaging's perceived benefits and challenges, offering a comprehensive overview of its influence on teaching practices and student outcomes.

Theme	Response Summary	Frequency (n=120)	Percentage (%)
**Perception of Translanguaging Practice**	Natural and beneficial strategy (consistent with high mean scores on perceived benefits items A1, A2, A9)	108	90.0
	Neutral	10	8.3
	Not beneficial	2	1.7
**Personal Use of Translanguaging**	Yes, to facilitate understanding and flow (aligns with high mean scores on usage items B17, B22)	102	85.0
	Sometimes, when necessary	15	12.5
	No, prefer using only English	3	2.5
**Intentionality of Translanguaging**	Intentionally (consistent with intentional usage to reduce anxiety and boost confidence items A9, A10)	96	80.0
	Both intentionally and unconsciously	18	15.0
	Unconsciously	6	5.0

Theme	Response Summary	Frequency (n=120)	Percentage (%)
**Situations for Translanguaging**	When struggling with grammar and vocabulary, discussing unfamiliar topics, explaining complex concepts	110	91.7
	Rarely, only when absolutely necessary	10	8.3
**Classroom Environment Contributions**	Positive contributions: increased participation, comfort in expression, and interaction (aligns with benefits items C24, C23)	105	87.5
	Neutral	12	10.0
	Negative contributions	3	2.5
**Language Acquisition Contributions**	Greatly contributes: better understanding, increased confidence, and motivation (consistent with perceived benefits items A9, A10, C24)	100	83.3
	Some contributions	18	15.0
	No contributions	2	1.7
**Possible Disadvantages of Translanguaging**	Reduced reliance on English, negative impact on proficiency, overuse (consistent with concerns noted in A3, A5)	85	70.8
	Few disadvantages	30	25.0

Theme	Response Summary	Frequency (n=120)	Percentage (%)
	No disadvantages	5	4.2
**Use of Different Languages in English Classes**	Enriches classroom environment helps understand cultural differences (aligns with benefits items C23, C27)	108	90.0
	Neutral	10	8.3
	Not beneficial	2	1.7
**Classroom and Cultural Contributions of Multilingualism**	Significant contributions: better understanding of different cultures, increased respect for cultural differences (consistent with benefits items C27, C26)	106	88.3
	Some contributions	12	10.0
	No contributions	2	1.7
**Impact on Promoting Multiculturalism and Multilingualism**	An essential role in promoting multiculturalism and multilingualism: improved communication skills and increased cultural awareness (aligns with benefits items C24, C23, C27)	109	90.8
	Some impact	10	8.3
	No impact	1	0.9

The data reveals a strong alignment between quantitative and qualitative findings, highlighting the overall positive perception and intentional use of translanguaging among pre-service EFL teachers. An overwhelming 90% of interviewees consider translanguaging a natural and beneficial strategy, consistent with high mean scores on perceived benefits (e.g., A1, A2, A9). A significant 85% use translanguaging to facilitate understanding and flow, aligning with high usage scores (e.g., B17, B22), while 80% employ it intentionally to reduce anxiety and boost confidence, mirroring related questionnaire items (e.g., A9, A10). Situationally, 91.7% use translanguaging when grappling with grammar, vocabulary, or complex concepts. Positive classroom contributions, including increased participation and comfort, were noted by 87.5%, aligning with benefits items (C24, C23). 83.3% acknowledged substantial benefits for language acquisition, resonating with related items (A9, A10, C24). However, 70.8% expressed concerns about over-reliance on native languages potentially impacting English proficiency, reflecting concerns noted in items (A3 and A5). Additionally, 90% believe using different languages enriches the classroom environment and aids cultural understanding, and 88.3% see significant contributions to cultural awareness, aligning with questionnaire findings (C23, C27). Finally, 90.8% view translanguaging as crucial for promoting multiculturalism and multilingualism, consistent with high scores on related benefits (C24, C23, C27). Below are sample student sentences for each theme to help illustrate their perspectives:

Perception of Translanguaging Practice

> *"I find translanguaging a very natural and helpful part of my learning. It makes it easier to understand complex topics."*
>
> *"For me, translanguaging is okay but only sometimes necessary. I can get by without it."*

"I do not see much benefit in using different languages in class. I prefer sticking to English."

Personal Use of Translanguaging

"I regularly use translanguaging in my studies to make sure I better grasp the meaning of new concepts."

"I use it only when I am stuck. Otherwise, I try to stick to English."

"I prefer to use only English in my classes, as I feel it is more effective."

Intentionality of Translanguaging

"I intentionally use translanguaging to ease my anxiety and boost my confidence in speaking English."

"Sometimes I use translanguaging without thinking about it, but other times I do it intentionally to help with my learning."

"I use different languages unconsciously whenever it happens during my learning process."

Situations for Translanguaging

"I mostly use translanguaging when dealing with difficult grammar rules or unfamiliar vocabulary."

"I only use translanguaging if I have to, usually in particular situations."

"I rarely use translanguaging unless necessary for understanding something."

Classroom Environment Contributions

"Translanguaging has improved my class participation and made me feel more comfortable expressing myself."

"I do not notice much difference in the classroom environment when translanguaging is used."

"In my experience, translanguaging sometimes makes the classroom environment less focused."

Language Acquisition Contributions

"Using translanguaging has greatly helped my understanding of English and made me feel more confident."

"Translanguaging helps with my language learning, but not as much as I had hoped."

"I do not feel that translanguaging contributes much to my language acquisition."

Possible Disadvantages of Translanguaging

"I worry that relying on translanguaging too much might weaken my English skills over time."

There are a few disadvantages, like potentially confusing the languages or relying on them too much.

"I do not see any real disadvantages in using translanguaging."

Use of Different Languages in English Classes

"Incorporating different languages in our English classes has made the environment richer and more inclusive."

"I feel neutral about using other languages in class; it does not make much difference to me."

"Using different languages does not seem to benefit my learning experience in English."

Classroom and Cultural Contributions of Multilingualism

"Multilingualism has significantly helped me understand and respect different cultures better."

"There are some benefits to multilingualism, but they are not as impactful as I expected."

"I do not see how multilingualism contributes much to my understanding of other cultures."

Impact on Promoting Multiculturalism and Multilingualism

"Translanguaging has played a major role in improving my communication skills and understanding of different cultures."

"It has some impact on multiculturalism and multilingualism, but not as much as I would have liked."

"I have not noticed much of an impact on promoting multiculturalism or multilingualism from translanguaging."

Table 10. Comparison of the Questionnaire and Interview Findings

Theme	Questionnaire Findings	Interview Findings
**Perception of Translanguaging Practice**	Generally positive, with high mean scores on items such as translanguaging being a natural practice (mean = 3.9501) and	Consistently positive, 90% of interviewees consider

	beneficial strategy (mean = 3.7681).	translanguaging a natural and beneficial strategy.
**Personal Use of Translanguaging**	There is high agreement on using translanguaging to give instructions (mean = 4.0748) and explain concepts (mean = 4.0599).	85% of interviewees stated they use translanguaging to facilitate understanding and flow.
**Intentionality of Translanguaging**	Mixed responses, with intentional use, were noted for reducing anxiety and boosting confidence (mean = 3.7307, 3.5137).	80% indicated intentional use, aligning with the questionnaire findings of using translanguaging to reduce anxiety and boost confidence.
**Situations for Translanguaging**	It was used mainly when giving instructions, explaining concepts, and helping with academic achievement (mean = 3.4264).	91.7% reported using translanguaging when struggling with grammar, vocabulary, or explaining complex concepts.
**Classroom Environment Contributions**	There is high agreement that translanguaging increases participation and motivation (mean = 4.0150, 4.0125).	87.5% stated positive contributions to participation, comfort, and interaction.

**Language Acquisition Contributions**	She was seen as beneficial for understanding and confidence (mean = 3.7307, 3.5137).	83.3% mentioned significant contributions to understanding, confidence, and motivation.
**Possible Disadvantages of Translanguaging**	Concerns about over-reliance on native languages impacting English proficiency (mean = 3.1272).	70.8% noted reduced reliance on English and a potential negative impact on proficiency.
**Use of Different Languages in English Classes**	There is moderate agreement on using translanguaging to develop language skills and conduct research (mean = 3.3541, 3.3142).	90% believed using different languages enriches the classroom environment and helps understand cultural differences.
**Classroom and Cultural Contributions of Multilingualism**	Recognized for increasing participation and ensuring effective communication (mean = 3.7382, 3.7207).	88.3% highlighted contributions to understanding different cultures and increasing respect for cultural differences.
**Impact on Promoting Multiculturalism and Multilingualism**	It fosters creative thinking and higher-order thinking skills (mean = 3.6833, 3.4713).	90.8% affirmed the role of translanguaging in promoting multiculturalism and multilingualism, improving communication skills, and increasing cultural awareness.

The comparison underscores a strong alignment between the quantitative and qualitative findings in the study on translanguaging practices among pre-service EFL teachers. The questionnaire and interview data consistently reveal positive perceptions of translanguaging as a beneficial and natural strategy in English language classrooms. The agreement on the intentional use of translanguaging to reduce anxiety and boost confidence and its significant contributions to participation, motivation, and understanding is evident across both datasets. Additionally, concerns about over-reliance on native languages and its potential impact on English proficiency are reflected in both sources. The qualitative interviews provide detailed contexts that complement the broader categories captured in the quantitative data, reinforcing the overall coherence and depth of the study's findings on the role and impact of translanguaging in fostering a multilingual and culturally rich classroom environment.

7. Discussion

The present study explored pre-service EFL teachers' perceptions of, and attitudes towards, translanguaging strategies, reported usage of these strategies in various instructional contexts, and the perceived benefits of translanguaging for English learners. The results provide valuable insights into how translanguaging is perceived and utilized in language education and how these findings compare with existing literature. The observed gender differences in perceptions of translanguaging among pre-service EFL teachers, as indicated by the t-test results, suggest that female participants generally hold more favourable views on translanguaging than their male counterparts across the scale and all its subscales. Several factors might contribute to these gender differences. Research suggests that women tend to have more collaborative and inclusive communication styles (Peck, 2016; Wood & Inman, 1993). Translanguaging, which promotes using multiple languages to facilitate understanding and learning, might align more closely with these collaborative tendencies (Ticheloven et al., 2021). This could make female pre-service teachers more open to and appreciative of translanguaging

practices. Women may experience different levels of language anxiety and confidence compared to men (Geçkin, 2020). Female participants might find that translanguaging reduces anxiety and increases confidence, reflected in their more positive perceptions (Dryden et al., 2021). Differences in educational experiences and exposure to language learning methods could influence perceptions (Brown, 2009; Orcasitas-Vicandi & Leonet, 2022). If female students have had more positive experiences with multilingual education or have been more frequently encouraged to use multiple languages, they may view translanguaging more favourably (Al-Bataineh & Gallagher, 2021; Gorter & Arocena, 2020). Female teachers might emphasize empathy and inclusivity in their teaching approaches (Demetriou et al., 20099). Translanguaging can be seen as an inclusive strategy that accommodates diverse linguistic backgrounds (Beatty et al., 2021), which may resonate more with female teachers' educational philosophies. Cultural and social factors can shape attitudes toward language learning and teaching (Jabeen & Shah, 2011). Gender roles and expectations within a specific cultural context might influence how male and female teachers perceive and value translanguaging practices (Sato, 2018; Yuvayapan, 2019). Differences in pedagogical beliefs between genders could also play a role (Richardson, 1996). Female teachers might be more inclined towards progressive teaching methods (Perrillo, 2004) that emphasize student-centred learning and the incorporation of students' linguistic and cultural backgrounds into the classroom (Baeten et al., 2016; Keller, 2018). The significant differences observed in the perceptions of translanguaging among different academic year groups, as detailed in Tables 4 and 5, can be attributed to several factors related to the educational journey and experiences of pre-service EFL teachers. Fourth-year students have had more extensive exposure to both pedagogical theories and practical teaching experiences. This increased exposure may lead to a deeper understanding and more favourable perceptions of translanguaging as an effective teaching strategy (Escobar, 2019). Senior students have more opportunities to apply theoretical knowledge in real classroom

settings (Brundiers et al., 2010), which can positively influence their perceptions if they observe the benefits of translanguaging first-hand. Third and second-year students are in the middle of their training, balancing theoretical knowledge with initial practical experiences (Watzke, 2007). They may still develop their confidence and understanding of effective teaching strategies, which might explain their lower mean scores than fourth-year students. Second and third-year Students are often at a critical transition point where they face more challenging coursework and practical teaching assignments (Walsch, 2009). This period might lead to a more critical or cautious view of translanguaging as they balance theoretical knowledge with classroom management and language teaching realities. Preparatory and first-year students may approach their studies enthusiastically and open-mindedly (Erickson et al., 2009). Their relatively high mean scores in subscale A could reflect an initial excitement about innovative teaching strategies like translanguaging, even if they have less practical experience. Early-stage students may hold idealistic views about language teaching methodologies (Rust, 1994; Warford & Reeves, 2003), including translanguaging, without encountering the practical challenges that more experienced students might face. In brief, the differences in perceptions of translanguaging among different academic year groups can be understood through the lens of pedagogical development, practical experience, and evolving educational perspectives. The findings highlight the importance of continuous exposure to and engagement with innovative teaching strategies throughout the teacher education program to foster a balanced, well-informed view of translanguaging.

The data indicate that pre-service EFL teachers hold a generally positive view of translanguaging. They perceive it as a natural practice (mean = 3.9501, SD = 1.15651) and beneficial strategy (mean = 3.7681, SD = 0.96363) for language learning. This aligns with García and Wei’s (2014) assertion that translanguaging is increasingly recognized as a valuable pedagogical tool that reflects and respects the multilingual realities of learners. Furthermore,

teachers believe translanguaging aids in reducing anxiety (mean = 3.7307, SD = 1.01601) and boosting confidence (mean = 3.5137, SD = 1.16423), supporting Auerbach's (1993) findings that translanguaging can lower language anxiety and increase learners' confidence by creating a more inclusive and supportive environment. Interestingly, there is less agreement on the notion that translanguaging indicates deficiencies in English language skills (knowledge mean = 3.1272, SD = 1.16029; speaking ability mean = 3.2818, SD = 1.16529; confidence mean = 3.0848, SD = 1.19908). This contrasts with traditional views in the literature, which have often seen translanguaging as a sign of language inadequacy (Cummins, 2019). Recent studies, however, argue that translanguaging should be viewed as a strategic choice rather than a deficiency (Hornberger & Link, 2012), which is consistent with the positive perceptions reported in this study. The study reveals that pre-service EFL teachers value translanguaging for giving instructions (mean = 4.0748, SD = 0.99468) and explaining concepts (mean = 4.0599, SD = 0.93883). These findings support Lewis, Jones, and Baker's (2012) assertion that translanguaging effectively clarifies complex content and provides instructional clarity. Moderate agreement was found regarding using translanguaging to assist students with academic achievement (mean = 3.4264, SD = 1.28460) and to develop foreign language skills (mean = 3.3890, SD = 1.15900). This aligns with Creese and Blackledge's (2010) observation that while translanguaging supports learning, its effectiveness can vary depending on the instructional context and student needs. The lower agreement on using translanguaging to support students facing difficulties (mean = 3.1621, SD = 1.31763) may indicate a gap between theoretical benefits and practical implementation. Poza (2017) similarly found that while translanguaging is advocated for, its application in supporting struggling students can be inconsistent, reflecting a potential area for further exploration and development. The benefits of translanguaging strategies were strongly endorsed for increasing participation (mean = 4.0150, SD = 1.01969) and motivation (mean = 4.0125, SD = 1.13681). This finding is

consistent with MacSwan's (2017) research, which highlights translanguaging's role in enhancing learner engagement and motivation. Moderate agreement was observed for translanguaging's effectiveness in debates (mean = 3.7382, SD = 1.11524), ensuring effective communication (mean = 3.7207, SD = 1.13216), and fostering creative thinking (mean = 3.6833, SD = 1.14103). These results corroborate Garcia's (2009) emphasis on translanguaging's utility in facilitating clearer communication and supporting creative processes. However, the lower agreement on translanguaging's role in enhancing critical and reflective thinking (mean = 3.4713, SD = 1.21441) is noteworthy. This finding suggests that while translanguaging is valued for participation and motivation, its impact on higher-order thinking skills might be less direct or less apparent. This observation aligns with Baker's (2011) research, which indicates that the benefits of translanguaging on cognitive skills may require further investigation. Overall, the study reveals a positive perception of translanguaging among pre-service EFL teachers, with high value placed on its role in instruction and learner engagement. These findings are consistent with recent literature that acknowledges translanguaging as a beneficial pedagogical strategy. However, the moderate to lower agreement on certain aspects, such as its impact on struggling students and higher-order thinking skills, suggests areas for further research. Future studies could explore these dimensions in greater depth to better understand the full scope of translanguaging's benefits and applications in diverse educational contexts.

8. Conclusion

The findings of this study highlight the generally positive perception of translanguaging among pre-service EFL teachers, demonstrating its recognized benefits in facilitating language learning, reducing anxiety, and boosting confidence. The significant gender differences, with female participants showing more favourable views, suggest possible influences from collaborative communication styles and different levels of language anxiety. Additionally, the

variations in perceptions across academic year groups indicate that more experienced students value translanguaging more highly, likely due to greater exposure to practical teaching contexts. Despite these positive perceptions, there is moderate agreement on using translanguaging to support struggling students and enhance higher-order thinking skills, indicating areas for further exploration. This study underscores the importance of continuous engagement with innovative teaching strategies like translanguaging in teacher education programs. It also suggests the need for more in-depth research into its varied impacts on language learning.

9. Implications

The findings of this study have significant implications for English language education and teacher training programs. The positive perceptions of translanguaging among pre-service EFL teachers underscore its potential as a valuable pedagogical strategy that can enhance language learning by reducing anxiety, increasing confidence, and fostering greater classroom participation. This suggests that integrating translanguaging practices into teacher training programs could better prepare future educators to support multilingual classrooms effectively. Additionally, the concerns about over-reliance on native languages and its potential impact on English proficiency highlight the need for balanced approaches in translanguaging practices. Educational policymakers and curriculum developers should consider these insights when designing language teaching frameworks and providing professional development opportunities. Furthermore, the positive contributions of translanguaging to cultural understanding and multiculturalism emphasize its role in promoting inclusive education and respect for diverse linguistic backgrounds. Overall, this study advocates for the thoughtful implementation of translanguaging strategies to maximize their benefits while addressing potential challenges, thereby enhancing the overall effectiveness of language education in multilingual contexts.

References

Al-Bataineh, A., & Gallagher, K. (2021). Attitudes towards translanguaging: How future teachers perceive the meshing of Arabic and English in children’s storybooks. International Journal of Bilingual Education and Bilingualism, 24(3), 386-400.

Ataş, U. (2023). Translanguaging in English-medium instruction (EMI): Examining English literature content classrooms. Turkish Journal of Education, 12(3), 142-157.

Auerbach, E. (1993). Reexamining English Only in the ESL Classroom. TESOL Quarterly, 27/1: 9-32.

Back, M., Han, M., & Weng, S. C. (2020). Emotional scaffolding for emergent multilingual learners through translanguaging: Case stories. Language and education, 34(5), 387-406.

Baeten, M., Dochy, F., Struyven, K., Parmentier, E., & Vanderbruggen, A. (2016). Student-centred learning environments: an investigation into student teachers’ instructional preferences and approaches to learning. Learning Environments Research, 19, 43-62.

Baker, C. (2011). Foundations of bilingual education and bilingualism (5th ed.). Bristol, UK: Multilingual Matters.

Barahona, M. (2020). The potential of translanguaging as a core teaching practice in an EFL context. System, 95, 102368.

Beatty, L., Acar, S., & Cheatham, G. A. (2021). Translanguaging in inclusive classrooms: Learning with children and families. Young Exceptional Children, 24(3), 154-169.

Bonacina-Pugh, F., da Costa Cabral, I., & Huang, J. (2021). Translanguaging in education. Language Teaching, 54(4), 439-471.

Brown, A. V. (2009). Students' and teachers' perceptions of effective foreign language teaching: A comparison of ideals. The Modern Language Journal, 93(1), 46-60.

Brundiers, K., Wiek, A., & Redman, C. L. (2010). Real-world learning opportunities in sustainability: from the classroom into the real world. International Journal of Sustainability in Higher Education, 11(4), 308-324.

Carstens, A. (2016). Translanguaging as a vehicle for L2 acquisition and L1 development: students' perceptions. Language Matters, 47(2), 203-222.

Cenoz, J., & Gorter, D. (2020). Pedagogical translanguaging: An introduction. System, 92, 102269.

Creese, A., & Blackledge, A. (2010). Translanguaging in the bilingual classroom: A pedagogy for learning and teaching? The Modern Language Journal, 94(1), 103-115.

Cummins, J. (2019). The emergence of translanguaging pedagogy: A dialogue between theory and practice. Journal of Multilingual Education Research, 9(13), 19-36.

Cummins, J. (2021). Rethinking the education of multilingual learners: A critical analysis of theoretical concepts (Vol. 19). Bristol: Multilingual Matters.

Demetriou, H., Wilson, E., & Winterbottom, M. (2009). The role of emotion in teaching: Are there differences between male and female newly qualified teachers' approaches to teaching? Educational studies, 35(4), 449-473.

Duarte, J. (2019). Translanguaging in mainstream education: A sociocultural approach. International Journal of Bilingual Education and Bilingualism, 22(2), 150-164.

Dryden, S., Tankosić, A., & Dovchin, S. (2021). Foreign language anxiety and translanguaging as an emotionally safe space: Migrant English as a foreign language learners in Australia. System, 101, 102593.

Escobar, C. F. (2019). Translanguaging by design in EFL classrooms. Classroom Discourse, 10(3-4), 290-305.

Erickson, B. L., Peters, C. B., & Strommer, D. W. (2009). Teaching first-year college students. New York: John Wiley & Sons.

Fang, F., Zhang, L. J., & Sah, P. K. (2022). Translanguaging in language teaching and learning: Current practices and future directions. RELC Journal, 53(2), 305-312.

Fang, F., & Liu, Y. (2020). 'Using all English is not always meaningful': Stakeholders' perspectives on the use of and attitudes towards translanguaging at a Chinese university. Lingua, 247, 102959.

Galante, A. (2020). Pedagogical translanguaging in a multilingual English program in Canada: Student and teacher perspectives of challenges. System, 92, 102274.

García, O. (2009). Bilingual education in the 21st century: A global perspective. Malden/Oxford: Wiley/Blackwell.

García, O., & Seltzer, K. (2016). The translanguaging current in language education. Flerspråkighet som resurs, 31, 19-30.

García, O., & Kleyn, T. (2016). Translanguaging theory in education. In Translanguaging with multilingual students (pp. 9-33). New York: Routledge.

García, O., & Wei, L. (2014). Translanguaging: Language, bilingualism and education. London: Palgrave Macmillan.

Geckin, V. (2020). Do gender differences affect foreign language anxiety and preferences for oral corrective feedback? Journal of Theoretical Educational Science, 13(3), 591-608.

Genc, E., Yüksel, D., & Curle, S. (2023). Lecturers' Translanguaging Practices in English-taught Classes in Turkey. *Journal of Multilingual Theories and Practices*, 4, 8-31

Gorter, D., & Arocena, E. (2020). Teachers' beliefs about multilingualism in a course on translanguaging. System, 92, 102272.

Holdway, J., & Hitchcock, C. H. (2018). Exploring ideological becoming in professional development for teachers of multilingual learners: Perspectives on translanguaging in the classroom. Teaching and Teacher Education, 75, 60-70.

Hornberger, N. H., & Link, H. (2012). Translanguaging and transnational literacies in multilingual classrooms: A biliteracy lens. International journal of bilingual education and bilingualism, 15(3), 261-278.

Jabeen, F., & Shah, S. K. (2011). The role of culture in ELT: Learners' attitude towards the teaching of target language culture. European Journal of Social Sciences, 23(4), 604-613.

Jaspers, J. (2018). The transformative limits of translanguaging. Language & Communication, 58, 1-10.

Jonsson, C. (2017). Translanguaging and ideology: Moving away from a monolingual norm. New perspectives on translanguaging and education, 108, 20-37.

Jones, B. (2017). Translanguaging in bilingual schools in Wales. Journal of Language, Identity & Education, 16(4), 199-215.

Karabulut, A., & Dollar, Y. K. (2022). The use of translanguaging pedagogy in writing classes of Turkish EFL learners. Participatory Educational Research, 9(6), 41-65.

Keiler, L. S. (2018). Teachers' roles and identities in student-centered classrooms. International journal of STEM education, 5, 1-20.

Khair, A. U., Rosmayanti, V., & Firman, A. (2020). Translanguaging pedagogy in promoting higher order thinking skill (HOTS) in Indonesian higher education. Asian EFL Journal, 27(2.3), 259–287.

Koçer, P. (2023). Translanguaging pedagogy: An integrative review study questioning its potential benefits and main concerns. The Literacy Trek, 9(3), 29-48.

Lewis, G., Jones, B., & Baker, C. (2012). Translanguaging: Origins and development from school to street and beyond. Educational research and evaluation, 18(7), 641-654.

Liu, Y., & Fang, F. (2022). Translanguaging theory and practice: How stakeholders perceive translanguaging as a practical theory of language. RELC Journal, 53(2), 391-399.

Leung, C., & Valdés, G. (2019). Translanguaging and the transdisciplinary framework for language teaching and learning in a multilingual world. The Modern Language Journal, 103(2), 348-370.

MacSwan, J. 2017. A Multilingual perspective on translanguaging. American Educational Research Journal 54. 167–201.

Makarova, I., Duarte, J., & Huilcán, M. I. (2023). Experts' views on the contribution of language awareness and translanguaging for minority language education. Language Awareness, 32(1), 74-93.

Makalela, L. (2015). Translanguaging as a vehicle for epistemic access: Cases for reading comprehension and multilingual interactions. Per Linguam: A Journal of Language Learning= Per Linguam: Tydskrif vir Taalaanleer, 31(1), 15-29.

Mwinda, N., & Van der Walt, C. (2015). From English-only to translanguaging strategies: exploring possibilities. Per Linguam: a Journal of Language Learning= Per Linguam: Tydskrif vir Taalaanleer, 31(3), 100-118.

Nagy, T. (2018). On translanguaging and its role in foreign language teaching. Acta Universitatis Sapientiae, Philologica, 10(2), 41-53.

Nursanti, R. R. (2021). Classroom strategies through translanguaging for multilingualism students. English Learning Innovation, 2(1), 17-27.

Orcasitas-Vicandi, M., & Leonet, O. (2022). The study of language learning in multilingual education: students' perceptions of their language learning experience in Basque, Spanish and English. International Journal of Multilingualism, 19(1), 124-141.

Ossa Parra, M., & Proctor, C. P. (2021). Translanguaging to understand language. tesol Quarterly, 55(3), 766-794.

Öztürk, E., Müller, O., & Brown, E. (2023). Bilingualism in the Classroom: Exploring Teachers' Beliefs, Attitudes, and Practices. Research Studies in English Language Teaching and Learning, 1(1), 35-43.

Palmer, D. K., Martínez, R. A., Mateus, S. G., & Henderson, K. (2014). Reframing the debate on language separation: Toward a vision for translanguaging pedagogies in the dual language classroom. The Modern Language Journal, 98(3), 757-772.

Peck, J. J. (2016). Women and promotion: The influence of communication style. In Gender and communication at work (pp. 50-66). Routledge.

Perrillo, J. (2004). Beyond "progressive" reform: Bodies, discipline, and the construction of the professional teacher in interwar America. History of Education Quarterly, 44(3), 337-363.

Poza, L. (2017). Translanguaging: Definitions, implications, and further needs in burgeoning inquiry. Berkeley Review of Education, 6(2).

Prilutskaya, M. (2021). Examining pedagogical translanguaging: A systematic review of the literature. Languages, 6(4), 180.

Richardson, V. (1996). The role of attitudes and beliefs in learning to teach. Handbook of research on teacher education, 2(102-119), 273-290.

Robillos, R. J. (2023). Implementing translanguaging with pedagogical approach in an English as a foreign language (EFL) listening classroom. International Journal of Instruction, 16(2), 623-642.

Rust, F. O. C. (1994). The first year of teaching: It's not what they expected. Teaching and teacher education, 10(2), 205-217.

Sato, E. (2018). Constructing Women's Language and Shifting Gender Identity through Intralingual Translanguaging. Theory and practice in language studies, 8(10), 1261-1269.

Shawaqfeh, A. T., Khasawneh, A. J., Jadallah Abed Khasawneh, Y., & Khasawneh, M. A. S. (2024). Unlocking Linguistic Diversity: Translanguaging in Jordanian Classrooms and Its Impact on Student Engagement and Academic Achievement. Migration Letters, 21(S2), 479-491.

Smith, H., & Robertson, L. (2020). SCT and translanguaging-to-learn: proposed conceptual integration. Language and sociocultural theory, 6(2), 213-233.

Sobkowiak, P. (2022). Translanguaging practices in the EFL classroom-the Polish context. Linguistics and Education, 69, 101020.

Song, J., Howard, D., & Olazabal-Arias, W. (2022). Translanguaging as a strategy for supporting multilingual learners' social-emotional learning. Education sciences, 12(7), 475.

Ticheloven, A., Blom, E., Leseman, P., & McMonagle, S. (2021). Translanguaging challenges in multilingual classrooms: scholar, teacher and student perspectives. International Journal of Multilingualism, 18(3), 491-514.

Vaish, V. (2019). Challenges and directions in implementing translanguaging pedagogy for low achieving students. Classroom Discourse, 10(3-4), 274-289.

Villacañas de Castro, L. S. (2016). The pedagogical turn in the research on cross-language transfer: Re-thinking the Language Interdependence and the Language Threshold Hypotheses. Tejuelo (Trujillo), 2016, vol. 23, p. 201-227.

Yasar, Y., M., & Dikilitas, K. (2022). Translanguaging in the development of EFL learners' foreign language skills in Turkish context. Innovation in Language Learning and Teaching, 16(2), 176-190.

Yilmaz, T. (2021). Translanguaging as a pedagogy for equity of language minoritized students. International Journal of Multilingualism, 18(3), 435-454.

Yuvayapan, F. (2019). Translanguaging in EFL classrooms: Teachers' perceptions and practices. Journal of Language and Linguistic Studies, 15(2), 678-694.

Walsh, C., Larsen, C., & Parry, D. (2009). Academic tutors at the frontline of student support in a cohort of students succeeding in higher education. Educational Studies, 35(4), 405-424.

Warford, M. K., & Reeves, J. (2003). Falling into it: Novice TESOL teacher thinking. Teachers and teaching, 9(1), 47-65.

Watzke, J. L. (2007). Foreign language pedagogical knowledge: Toward a developmental theory of beginning teacher practices. The Modern Language Journal, 91(1), 63-82.

Wood, J. T., & Inman, C. C. (1993). In a different mode: Masculine styles of communicating closeness. Journal of Applied Communication Research, 21(3), 279-295.

Appendix

Perceptions of Translanguaging in English Lessons Survey

A. Demographic Information
Gender:
Grade:

Note: *Translanguaging is a strategy that involves using multiple languages in lessons. In this context, translanguaging can occur, and other known languages (e.g., Turkish, Azerbaijani, Kurdish, Arabic, English, etc.) can be used at any stage of the lesson.*

Statements	Strongly Disagree	Disagree	Neutral	Agree	Strongly Agree
**A1. Translanguaging is a natural practice in English lessons.**	**1**	**2**	**3**	**4**	**5**

Statements	Strongly Disagree	Disagree	Neutral	Agree	Strongly Agree
**A2. Translanguaging is a useful practice in English lessons.**	**1**	**2**	**3**	**4**	**5**
**A3. Translanguaging in English lessons is due to a lack of English language skills.**	**1**	**2**	**3**	**4**	**5**
**A4. Translanguaging in English lessons is due to a lack of English speaking skills.**	**1**	**2**	**3**	**4**	**5**
**A5. Translanguaging in English lessons is due to a lack of confidence in using English.**	**1**	**2**	**3**	**4**	**5**
**A6. Translanguaging in English lessons is due to anxiety about using English.**	**1**	**2**	**3**	**4**	**5**
**A7. Translanguaging is necessary for learning English.**	**1**	**2**	**3**	**4**	**5**
**A8. Translanguaging facilitates learning English.**	**1**	**2**	**3**	**4**	**5**
**A9. Translanguaging reduces my anxiety in English.**	**1**	**2**	**3**	**4**	**5**
**A10. Translanguaging increases my confidence in English.**	**1**	**2**	**3**	**4**	**5**
**A11. Translanguaging is a disadvantage for non-multilingual students in English classes.**	**1**	**2**	**3**	**4**	**5**
**A12. I view the use of different languages in English lessons positively.**	**1**	**2**	**3**	**4**	**5**
**A13. The use of different languages in English lessons raises my awareness about preserving these languages.**	**1**	**2**	**3**	**4**	**5**
**A14. I see the use of different languages in English lessons as part of human rights.**	**1**	**2**	**3**	**4**	**5**
**English teachers should use translanguaging in the following situations:**					
**B15. English teachers should use translanguaging to assist students with sufficient academic performance.**	**1**	**2**	**3**	**4**	**5**
**B16. English teachers should use translanguaging to further develop high-achieving students.**	**1**	**2**	**3**	**4**	**5**
**B17. English teachers should use translanguaging to explain unfamiliar concepts.**	**1**	**2**	**3**	**4**	**5**
**B18. English teachers should use translanguaging to improve foreign language skills.**	**1**	**2**	**3**	**4**	**5**

Statements	Strongly Disagree	Disagree	Neutral	Agree	Strongly Agree
**B19. English teachers should use translanguaging to give instructions to students.**	**1**	**2**	**3**	**4**	**5**
**B20. English teachers should use translanguaging to explain the topic.**	**1**	**2**	**3**	**4**	**5**
**B21. English teachers should use translanguaging to conduct research on the topic.**	**1**	**2**	**3**	**4**	**5**
**B22. English teachers should use translanguaging to support students where they struggle.**	**1**	**2**	**3**	**4**	**5**
**Using translanguaging benefits English students in the following ways:**					
**C23. Using translanguaging benefits English students by increasing participation.**	**1**	**2**	**3**	**4**	**5**
**C24. Using translanguaging benefits English students by increasing motivation.**	**1**	**2**	**3**	**4**	**5**
**C25. Using translanguaging benefits English students by engaging in debates.**	**1**	**2**	**3**	**4**	**5**
**C26. Using translanguaging benefits English students by ensuring effective communication.**	**1**	**2**	**3**	**4**	**5**
**C27. Using translanguaging benefits English students by developing creative thinking.**	**1**	**2**	**3**	**4**	**5**
**C28. Using translanguaging benefits English students by fostering critical and reflective thinking.**	**1**	**2**	**3**	**4**	**5**

Ethics Certificate

Click here to access/download
Other
renamed_365c2.pdf
